# A Two-Axis Goniometric Sensor for Tracking Finger Motion

**DOI:** 10.3390/s17040770

**Published:** 2017-04-05

**Authors:** Lefan Wang, Turgut Meydan, Paul Ieuan Williams

**Affiliations:** Wolfson Centre for Magnetics, School of Engineering, Cardiff University, Cardiff CF24 3AA, Wales, UK; WangL54@cardiff.ac.uk (L.W.); WilliamsPI1@cardiff.ac.uk (P.I.W.)

**Keywords:** finger motion tracking, angle measurement, flexion/extension, abduction/adduction, 3D printing, optical sensor, glove-based systems

## Abstract

The study of finger kinematics has developed into an important research area. Various hand tracking systems are currently available; however, they all have limited functionality. Generally, the most commonly adopted sensors are limited to measurements with one degree of freedom, i.e., flexion/extension of fingers. More advanced measurements including finger abduction, adduction, and circumduction are much more difficult to achieve. To overcome these limitations, we propose a two-axis 3D printed optical sensor with a compact configuration for tracking finger motion. Based on Malus’ law, this sensor detects the angular changes by analyzing the attenuation of light transmitted through polarizing film. The sensor consists of two orthogonal axes each containing two pathways. The two readings from each axis are fused using a weighted average approach, enabling a measurement range up to 180${}^{\circ }$ and an improvement in sensitivity. The sensor demonstrates high accuracy (±0.3${}^{\circ }$), high repeatability, and low hysteresis error. Attaching the sensor to the index finger’s metacarpophalangeal joint, real-time movements consisting of flexion/extension, abduction/adduction and circumduction have been successfully recorded. The proposed two-axis sensor has demonstrated its capability for measuring finger movements with two degrees of freedom and can be potentially used to monitor other types of body motion.

## 1. Introduction

**Figure 1:**
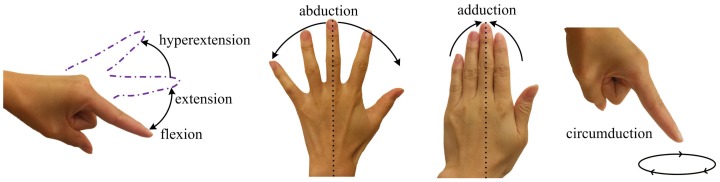
Finger motion: flexion, extension, abduction, adduction, and circumduction.

The hand is an amazing feat of human evolution enabling incredible physical dexterity and the ability to manipulate and develop tools through a series of finger motions, i.e., flexion, extension, abduction, adduction, and circumduction. Finger flexion is joint motion towards the palm relative to the standard anatomical position with extension being in the opposite direction as shown in Figure 1. Full extension is in line with the back of the hand and is defined as zero degree flexion. An injury can occur when joints are hyperextended too much [1]; in this paper, only hyperextension within normal limits is of interest. Finger abduction (ABD) and adduction (ADD) are the movements away from or towards the hand’s midline (dotted line), respectively. Circumduction is a circular motion combining flexion, extension, abduction and adduction [2].

Understanding and measuring hand kinematics is important in research areas such as biomechanics and medicine. It is a challenging subject due to the articulated nature of the hand and the associated multiple degrees of freedom (DOFs). Standard clinical goniometers are typically manual devices used to assess the range of joint movement in fingers [3,4]. This method is only effective for static measurements and usually requires a long time to fully assess the whole hand. The accuracy (±5${}^{\circ }$) is largely determined by the skill of the clinician or therapist; therefore, an accurate system that is able to monitor dynamic hand movements could be of significant benefit in clinical practice.

Various sensing techniques have been proposed to track finger motion, including resistive carbon/ink sensors [5,6], microfluidic strain sensors [7,8], magnetic induction coils [9], hetero-core spliced fibre sensors [10], fibre Bragg grating sensors [11,12] and multiple inertial measurement units (IMUs) [13,14]. Resistive film sensors are particularly favoured in glove-instrumented devices due to their flexibility, light weight, and low cost; however, they often suffer from instability, long transient response, and a high dependency on the radius of curvature [15], resulting in low accuracy. Previous work has shown commercial resistive bend sensors (Flexpoint Sensor Systems Inc., Draper, UT, USA [16]) suffer from severe hysteresis error and signal drift compared to our optical-based sensor [17,18]. In contrast, magnetic techniques are capable of monitoring precise hand movements, but the accuracy is significantly affected by interference from the Earth’s geomagnetic field or nearby ferromagnetic objects [19]. Optical fibre techniques measure finger flexion by detecting the attenuation of transmitted light as a function of fibre curvature; a polishing process [20] or the creation of imperfections [21] is required to increase sensitivity.

Using one or more of these sensing techniques, several glove-based devices have been developed to capture hand movements, e.g., SIGMA glove [22], SmartGlove [23], WU Glove [24], NeuroAssess Glove [25], Shadow Monitor [26], HITEG-Glove [27], Pinch Glove [28], Power Glove [29], CyberGlove [30], HumanGlove [31], and 5DT Data Glove [32]. These glove-based devices in most cases only detect finger flexion and/or extension using one-DOF sensors [10,22,25,27,33,34]. The measurement accuracy is usually adversely affected when monitoring two-DOF finger joints, i.e., the metacarpophalangeal (MCP) joints. The main reason is that finger ABD/ADD and flexion/extension do not occur in isolation [35], leading to unwanted deformations of the flexion sensors. Few glove systems have been able to measure two-DOF finger movements. One method is to use multiple one-DOF flexible sensors. The flexion/extension sensors are placed on the finger joints, and the ABD/ADD sensors are attached on the dorsal surface of the proximal phalanxes of adjacent fingers in an arched configuration [22,24,30,32]. In this approach, reliable ABD/ADD readings can only be obtained when the adjacent fingers have similar flexion; otherwise, twisting of the ABD/ADD sensor can occur, leading to inaccurate readings. Gestures, such as crossed fingers, are particularly prone to this problem. An alternative method is the use of IMUs that can provide 3D information of hand motion including finger ABD/ADD. The major drawback is the accumulated error since IMUs estimate the orientation trajectories by time integration of the inertial signal. Additionally, complicated models and data fusion processes are also required [14,36], causing large computation times for multiple joint articulations, thus limiting the system’s real-time capability.

Both finger flexion/extension and ABD/ADD are important parameters for finger kinetics. The majority of studies to date only consider finger flexion and extension, but accurate information on finger ABD/ADD is essential for areas such as medical diagnostics and rehabilitation. Accurate and simultaneous monitoring of two-DOF motion in human hand joints is an area underexplored in current glove-based systems. The main purpose of this work is to demonstrate a method for tackling this problem. We propose a two-axis optical sensor with an operating method based on Malus’ Law, capable of tracking the finger flexion/extension and ABD/ADD simultaneously. Additionally, the sensor demonstrates an ability to capture the finger circumduction, which is important for exploring the complex relationships between finger flexion/extension and ABD/ADD. This optical sensor features a wide measuring span of up to 180${}^{\circ }$ for each axis, which is sufficient for measuring the entire range of finger motion. In the following sections, the hand skeleton model, sensor principle and fabrication, quasi-static characteristics, as well as the recorded movements of the MCP joint of the left index finger will be described.

## 2. Hand Skeleton Model

The human hand is highly articulated, but constrained at the same time. Each hand has up to 27 DOFs, 21 of which are contributed by the five finger joints for local movements and the other six for global hand movements [35,37]. Each digit, except the thumb, possesses four DOFs, one each for the proximal interphalangeal (PIP) and distal interphalangeal (DIP) joints, and the other two for the MCP joint. The thumb has a more complicated structure, possessing five DOFs, one for the interphalangeal (IP) joint, two for the MCP joint, and two more for the carpometacarpal (CMC) joint [35].

Six types of synovial joints typically exist in the human body with differing structure and mobility [38] (pp. 18–20): plane joints, hinge joints, pivot joints, ellipsoidal joints, saddle joints, and ball and socket joints. Human fingers only have hinge, ellipsoid and saddle joints. The uniaxial and stable hinge joint allows flexion/extension only, performing back and forth movements. The PIP, DIP, and IP joints are examples of the hinge type [38] (pp. 170, 174). Ellipsoidal joints, also known as condyloid joints, consist of two oval articular surfaces; one is concave, and the other one is convex. This type of joint is able to move in two planes, allowing flexion, extension, abduction, adduction, and circumduction. MCP joints are examples of the ellipsoidal type [38] (p. 169). Saddle joints are also biaxial and perform a series of movements, similar to ellipsoidal joints, utilizing two reciprocally concave–convex surfaces. The CMC joint of the thumb is a typical saddle joint [38] (pp. 164–167).

In this paper, we focus on detecting the motion of the MCP joint. The range of motion varies between individuals [39], but, in general, flexion is around 90${}^{\circ }$, and slightly less for the index finger [35]. The active hyperextension of the index finger is approximately up to 30${}^{\circ }$. ABD/ADD generally have a greater range when the fingers are fully extended, being as much as 30${}^{\circ }$ in each direction [38] (p. 174).

## 3. Sensor Design

### 3.1. Principle of Operation

**Figure 2:**
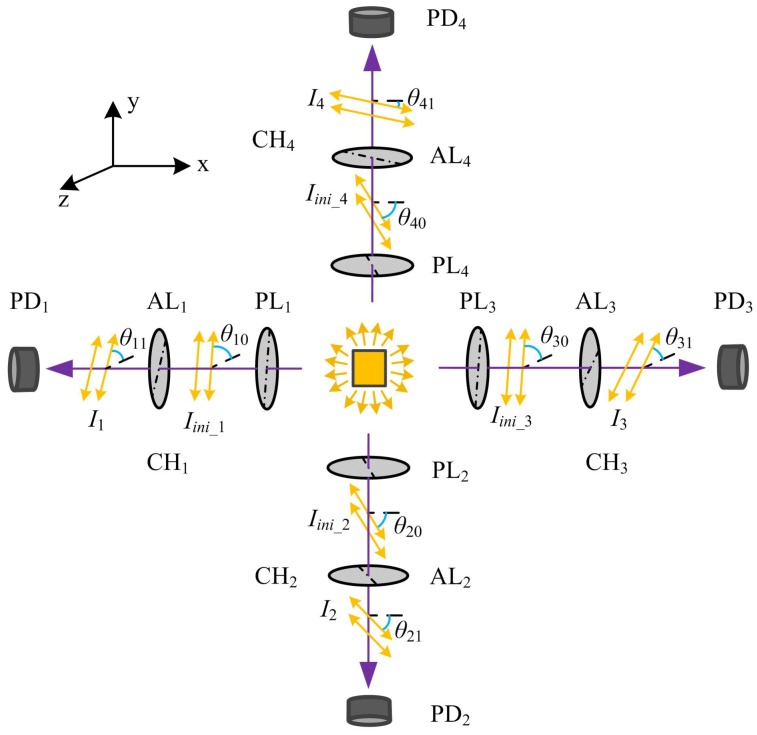
The schematic diagram of the optical sensor. PL${}_{i}$ and AL${}_{i}$ are linear polarizing films for the sensing channel CH ${}_{i}$ (*i* = 1, 2, 3, and 4). PD${}_{i}$ represents the photodiode which converts the light intensity into an electrical signal.

As illustrated in Figure 2, the optical sensor comprises four optical pathways, which, for ease of description, are now referred to as channels (CH${}_{i}$, *i* = 1, 2, 3, and 4). Each channel includes a linear polarizer, a polarizing film analyzer, and a photodiode. The unpolarized light source provides visible light for all four channels. Passing through the linear polarizer PL${}_{i}$, the incident light is partially blocked by the corresponding analyzer AL${}_{i}$ in CH${}_{i}$, and is recorded by the photodiode PD${}_{i}$. CH${}_{1}$ and CH${}_{3}$ are oriented along the *x*-axis, and CH${}_{2}$ and CH${}_{4}$ along the *y*-axis.

According to Malus’ Law, the final light intensity for each channel should obey the following theoretical relationship:

(1) $$I_{i}=I_{ini\_i}\cos^{2}\left(\theta_{i1}-\theta_{i0}\right),$$

where the subscript *i* denotes the channel number. *I${}_{i}$* denotes the detected light intensity, and *I${}_{ini\_i}$* represents the initial polarized intensity at θ${}_{i}$${}_{0}$ degrees. The parameter θ${}_{i}$${}_{1}$ denotes the deviated angle of the analyzer’s transmission axis from the *z*-axis (*i* = 1, 3) or *x*-axis (*i* = 2, 4) shown in Figure 2.

Clearly, the final intensity obtained for each channel is proportional to the angle between the transmission axes of the polarizer and the analyzer. For this optical sensor, the received intensity is linearly converted into electrical current, and then modulated and amplified by current-to-voltage conditioning circuitry. The circuits are similar to the ones presented in our previous work [18]. Finally, the measured voltage (*V${}_{i}$*) can be calculated by Equation (2).

(2) $$V_{i}=m_{i}I_{i}+b_{i}=m_{i}I_{ini\_i}\cos^{2}\left(\theta_{i1}-\theta_{i0}\right)+b_{i}=a_{i}\cos^{2}\delta_{i}+b_{i},\;\left(i=1,2,3,4\right),$$

where δ${}_{i}$ denotes the rotation angle of AL${}_{i}$ relative to the polarization direction of PL${}_{i}$. The parameters *m*${}_{i}$ and *a*${}_{i}$ are the constants of proportionality, and *b*${}_{i}$ is a constant voltage related to the extinction ratio of the polarizing films when set orthogonally.

From Equation (2), it can be found that a single channel only allows a limited measuring range of 90${}^{\circ }$ and the sensitivity is not constant. In order to track the entire flexion/extension of the MCP joints accurately (up to 120${}^{\circ }$ including active hyperextension), we use two channels CH${}_{1}$ and CH${}_{3}$ to capture the movements simultaneously along one axis, and then combine the measured data. Supposing that the transmission axes of the polarizers PL${}_{1}$ and PL${}_{3}$ are in the same direction, the analyzers AL${}_{1}$ and AL${}_{3}$ are initially oriented with their transmission axes at 45${}^{\circ }$ to each other. This ensures that at least one channel will be operating in the region with the maximum sensitivity and linearity. In fact, the oriented angle can be any value ranging from 40${}^{\circ }$ to 50${}^{\circ }$, since the cosine squared function demonstrates a high linearity in the range of 20${}^{\circ }$ to 70${}^{\circ }$. Additionally, CH${}_{2}$ and CH${}_{4}$ are used to capture the finger ABD/ADD movements, even though the ABD/ADD range is less than 90${}^{\circ }$. CH${}_{2}$ and CH${}_{4}$ are operated in the same way as CH${}_{1}$ and CH${}_{3}$.

### 3.2. Sensor Manufacture

The framework of the two-axis optical sensor is fabricated by an EnvisionTEC’s Perfactory Mini Multi Lens 3D Printer (EnvisionTEC, Gladbeck, Germany [40]) using the nanoparticle-filled material RCP30. RCP30 has excellent stiffness and opaqueness, making the sensor robust and improving interference immunity from ambient light sources.

**Figure 3:**
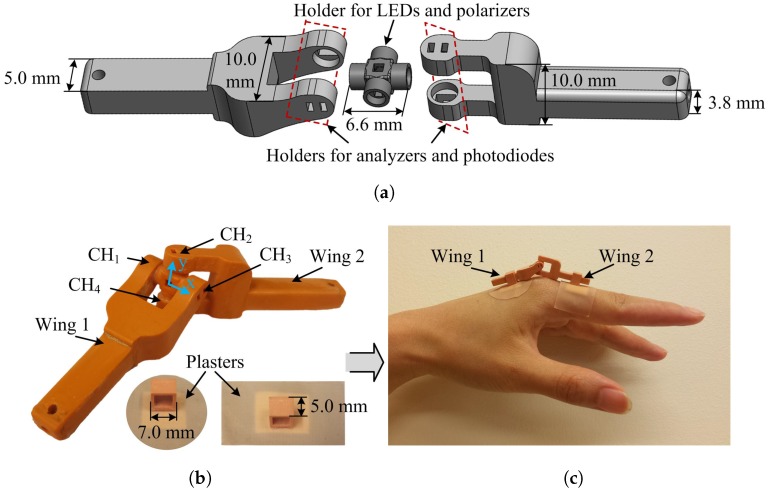
The two-axis optical sensor. (**a**) the 3D design produced in SolidWorks 2015 (DS SolidWorks Corp., Waltham, MS, USA); (**b**) a photograph of the complete optical sensor and the two rectangular hollow parts glued on the adhesive plasters; (**c**) an example of the sensor’s placement on the metacarpophalangeal joint of the left index finger.

Surface mounted LEDs (KPG-1608ZGC, Farnell element14, Leeds, UK [41]), with a dominant wavelength of 525 nm and a thickness of 0.25 mm, are employed as the light source. In this sensor, all of the polarizers and the analyzers are made from commercial linear polarizing sheet (Edmund Optics Ltd., York, UK [42]), with an extinction ratio of 9000:1 and high transmission from 400 nm to 700 nm. The photodiodes (TEMD6200FX01, Farnell element14, Leeds, UK [41]) with peak sensitivity at 540 nm are used to detect the resultant light intensities. As shown in Figure 3a, the LEDs and linear polarizers are located inside a cross-shaped holder that is optically aligned with the analyzers and photodiodes in the two wing sections.

Figure 3b shows a photograph of the complete two-axis optical sensor. When the sensor wings are aligned, the horizontal line along CH${}_{1}$ and CH${}_{3}$ is defined as the *x*-axis and the vertical line along CH${}_{2}$ and CH${}_{4}$ as the *y*-axis. The prototype sensor has an overall width of 10 mm, height of 10 mm and a length of 56 mm, which could be reduced with more advanced manufacturing methods. To eliminate the influence of ambient light, non-reflective black Aluminum foil tape (AT205, Thorlabs Ltd., Ely, UK [43]) was used to cover the optical sensor, which was not included in Figure 3b for clarity. This tape totally blocks out ambient light according to the manufacturer’s specification. For this study, the sensor was attached to the finger using rectangular hollow components and adhesive plasters as shown in Figure 3b.

An example of the optical sensor attached to the MCP joint of the index finger is shown in Figure 3c. The two rectangular components glued on the adhesive plasters are placed on the metacarpal and the proximal phalanx of the index finger. The sensor wings are inserted into the rectangular parts, enabling them to slide in response to finger movements. When the index finger flexes or extends, rotation of the wings occur about the *x*-axis of the sensor. Similarly, ABD/ADD motion will cause a rotation about the *y*-axis. The optical sensor is also able to record finger circumduction by combining data from both rotation directions.

## 4. Methods

### 4.1. Measurement Apparatus

**Figure 4:**
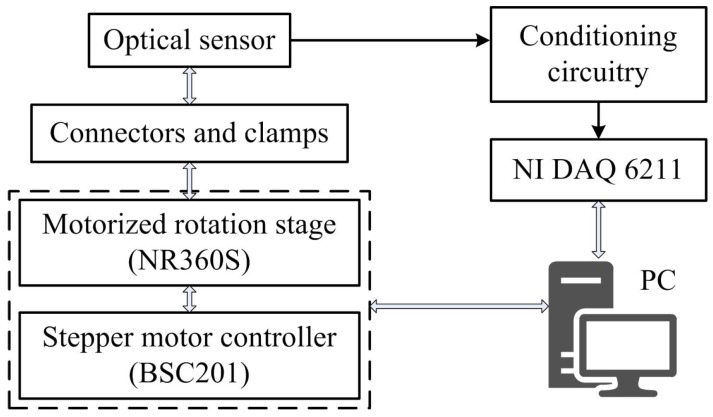
The block diagram of the automated measurement apparatus.

An automated experimental set-up, shown diagrammatically in Figure 4, is used to evaluate the performance of the optical sensor. A 360${}^{\circ }$ continuous motorized rotation stage (NR360S, Thorlabs Inc. [43]), with one arcsec resolution, controlled the rotation angles. The stepper motor offers an accuracy of up to 5 arcmin when driven by the micro-stepping controller (BSC201).

Each axis of the sensor was assessed separately using the same rotation stage. Evaluating the sensor rotation about the *x*-axis, Wing 2 was clamped rigidly and Wing 1 was connected to the central aperture of the stepper motor via a drill chuck. Similarly, for the rotation testing about the *y*-axis, Wing 1 was clamped and Wing 2 allowed to rotate under the control of the rotation stage. The resultant output signal was modulated by conditioning circuitry. The rotation system was controlled using ActiveX interfacing technology in LabVIEW 2014 (National Instruments, Newbury, UK) together with signal acquisition and processing using a multifunction DAQ (NI USB-6211, National Instruments).

### 4.2. Data Fusion Approach

**Figure 5:**
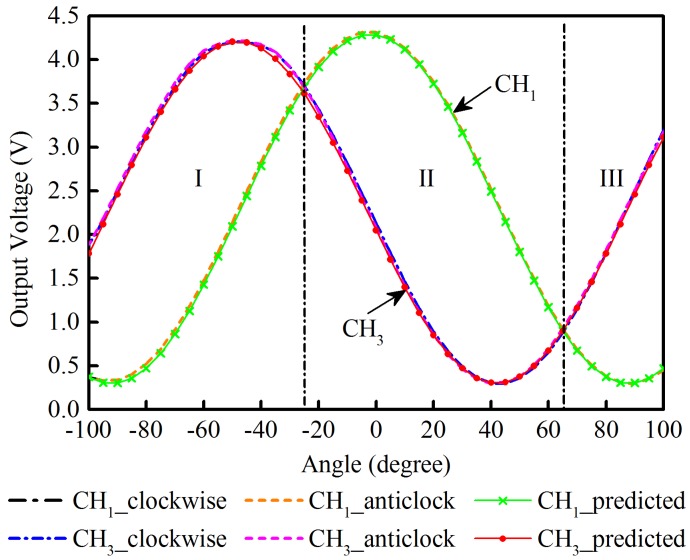
The voltage-to-angle relationships of the sensing channels CH${}_{1}$ and CH${}_{3}$ of the optical sensor. CH${}_{1}$_clockwise and CH${}_{1}$_anticlock represent the outputs of CH${}_{1}$ when the sensor rotates from −100${}^{\circ }$ to 100${}^{\circ }$, and then back to −100${}^{\circ }$. CH${}_{1}$_predicted is the predicted output value of channel CH${}_{1}$. Similarly, CH${}_{3}$_clockwise, CH${}_{3}$_anticlock, and CH${}_{3}$_predicted represent the corresponding readings of CH${}_{3}$.

To explain the data fusion method, rotation about the *x*-axis is used as an example, i.e., data acquired for CH${}_{1}$ and CH${}_{3}$. The amplitude of the output voltages obtained for CH${}_{1}$ and CH${}_{3}$, can be divided into three regions: I, II, and III, as shown in Figure 5. In region I and region III, the outputs of CH${}_{1}$ are smaller in comparison with CH${}_{3}$, while the opposite is true in region II. By comparing the voltage amplitudes from both channels, we can derive the rotation angle over a 180${}^{\circ }$ range using Equation (2). To avoid using data from one channel only, a weighted average method was used to calculate the angular position. Variable weighting was applied to each channel taking into account the angular dependency of their sensitivities. Higher weights were applied when the channel sensitivity was at its maximum, i.e., close to angles of 45${}^{\circ }$. Therefore, the final rotation angle about the *x*-axis, δ${}_{x}$, can be computed by Equation (3):

(3) $$\begin{array}{cc} \delta_{x} & =w_{1}\delta_{1}+w_{3}\delta_{3},\\ w_{1} & ={\left(1-\frac{|V_{1}-V_{1}\left(\delta_{1}=45\right)|}{|V_{1}-V_{1}\left(\delta_{1}=45\right)\left|+\right|V_{3}-V_{3}\left(\delta_{3}=45\right)|}\right)}^{n},\\ w_{3} & =1-w_{1}, \end{array}$$

where ω${}_{1}$ and ω${}_{3}$ denote the allocated different weights for CH${}_{1}$ and CH${}_{3}$, respectively. *V${}_{i}$* represents the output voltage proportional to the rotational angle δ${}_{i}$ in CH${}_{i}$ (*i* = 1, 3). The parameter *n* is determined by the nonlinear least squares regression based on the dataset explained in Section 5 and represented in Figure 5.

## 5. Sensor Characteristics

The quasi-static performance of the two-axis optical sensor is evaluated using the automated measurement apparatus at room temperature. The motor performs rotations at 10 degrees per second and the data is acquired at 100 Hz. The physical position when the two wings of the sensor are aligned is defined as the origin.

To investigate the sensor’s angle-to-voltage relationship, the optical sensor was rotated, about the *x*-axis, from −100${}^{\circ }$ to 100${}^{\circ }$ and then back to −100${}^{\circ }$ using increments of 5${}^{\circ }$. The data set was composed of 500 samples at each angle setting. This process was repeated five times with an interval of three minutes between each sampling cycle. The same procedure was carried out for the rotation testing about the *y*-axis.

By averaging the data from CH${}_{1}$ and CH${}_{3}$ at each angle over five cycles, the voltage-to-angle relationship of both channels was obtained for both clockwise and anticlockwise rotations. The angular dependence of the output voltages is plotted in Figure 5. According to Equation (2), *a${}_{i}$* and *b${}_{i}$* are the output span and the offset voltage (the minimum output) of each channel CH${}_{i}$ (*i* = 1, 3). The predicted values for CH${}_{1}$ and CH${}_{3}$ are calculated and also plotted in Figure 5. CH${}_{2}$ and CH${}_{4}$ have a similar performance to that of CH${}_{1}$ and CH${}_{3}$ and have been omitted from Figure 5 for clarity.

As seen in Figure 5, both channels provide readings consistent with the predicted values for bidirectional rotation. The phase difference between the waveforms is consistent with the difference in orientation of each channel.

The sensor’s output characteristics are listed in Table 1. It demonstrates consistent performance between each channel with an average deviation from the theoretical voltage, equal to ±2.3%. The overall hysteresis error is less than 1.7% for rotation in both directions and the repeatability, as determined from the averaged relative standard deviations, is equal to 0.5%.

In the linear region, where δ${}_{i}$ is in the range 20${}^{\circ }$ to 70${}^{\circ }$, the sensitivity of each channel is 62.6 mV/${}^{\circ }$± 0.9 mV/${}^{\circ }$; this can be further increased by adjusting the amplification factor of the conditioning circuits. According to the signal-to-noise level, the resolution is 0.1${}^{\circ }$ under laboratory conditions for the linear regions, comparing favorably with the 0.5${}^{\circ }$ achieved in commercial resistive bend sensors [25].

## 6. Sensor Performance Validation

### 6.1. Random Angle Testing

This investigation evaluated the efficiency of the data fusion approach and the measurement ability of the optical sensor. Using MATLAB 2014 (MathWorks, Natick, MA, USA), we generated one hundred uniformly distributed pseudorandom numbers to simulate finger motion. The angular data in the range of −70${}^{\circ }$ to 110${}^{\circ }$ was used to simulate the motion of an MCP joint in flexion or hyperextension, and the range was changed to −90${}^{\circ }$ to 90${}^{\circ }$ for the simulation of ABD/ADD movements. Following the rotation of the automated motorized system, the optical sensor rotated successively from one measurement position to the next, with five hundred samples recorded at each angle. The weighted average method was performed within LabVIEW and combines the outputs from CH${}_{1}$ and CH${}_{3}$ when monitoring the *x*-axis rotation and similarly combining CH${}_{2}$ and CH${}_{4}$ for *y*-axis monitoring.

The manufacturer’s design guide states that the motorized rotation stage has an accuracy of 0.08${}^{\circ }$. Over the one hundred samples of random angles, the angle measured by the optical sensor differed from the set motor angle by an average of ±0.2${}^{\circ }$ in the *x*-axis and ±0.3${}^{\circ }$ in the *y*-axis. The slight difference of the sensor performance about the two axes, as well as differences between individual channels (shown in Table 1), may be due to inconsistencies in fabrication.

Nevertheless, the overall mean absolute error of the optical sensor is less than 0.3${}^{\circ }$, and this is more accurate than some reported sensors: the linear potentiometer (0.7${}^{\circ }$) [44], the embedded hetero-core optic fibre sensor (0.9${}^{\circ }$) [10], the resistive bend sensor (1.5${}^{\circ }$) [45], and the fibre Bragg grating sensor (2.0${}^{\circ }$) [11].

### 6.2. Finger Motion Detection

To assess the sensor’s capability for monitoring finger motion, we attached the optical sensor on to the dorsal surface of a healthy volunteer’s left index finger in a similar fashion to that shown in Figure 3c. During these measurements, the participant is initially asked to sit with their arm relaxed and resting on a table. The position when the whole hand is flat is regarded as 0${}^{\circ }$ flexion/extension and where the fingers are closed is treated as 0${}^{\circ }$ ABD/ADD.

#### 6.2.1. Finger Flexion and Extension

The participant initially placed their hand flat on the table with fingers close together. After several seconds, the participant formed a fist by rapidly bending their fingers, then remaining at that position for a few seconds before extending the fingers to maximum hyperextension. This procedure was repeated continuously for five cycles. During this testing, the fingers (except the thumb) are always kept close to each other so that ABD/ADD can be regarded as 0${}^{\circ }$. Photographs were taken to record finger positions at their maximum flexion and hyperextension as a reference. For example, the angular motion of the left index finger was assessed by measuring the lines extrapolated from the photographs along the proximal phalange of the index finger and the back of the hand using the image processing program ImageJ (ver. ij150) [46].

**Figure 6:**
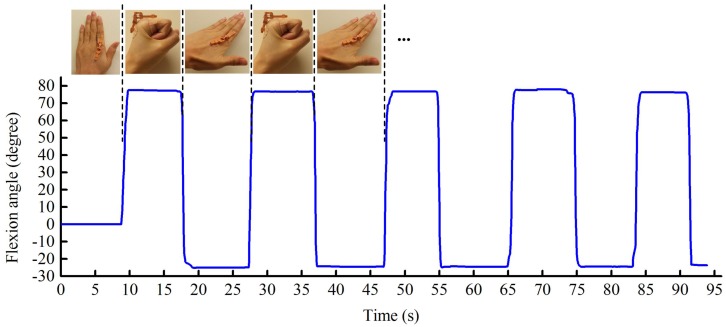
The repeated flexion and extension of the metacarpophalangeal joint of the participant’s left index finger.

Figure 6 demonstrates the dynamic response of the optical sensor to alternating finger flexion and extension. The positive readings represent index finger flexion or extension, and the negative angles indicate hyperextension. Variations in shape and width of the waveform are due to variations in the subject’s hand movements and periods of inactivity, respectively.

For this particular test subject, the optical sensor recorded a maximum finger flexion of 78.5${}^{\circ }$ when the hand was clenched into a fist, and a maximum hyperextension of 25.1${}^{\circ }$, and this compares to readings of 80.7${}^{\circ }$ and 22.9${}^{\circ }$ for measurements obtained from photographs.

#### 6.2.2. Finger Abduction and Adduction

This test involved measuring ABD/ADD, and as with the flexion test, the participant begins with the same open hand posture. Whilst maintaining the index finger in the same position, the other fingers were made to curl towards the palm. This was followed by the participant moving their index finger to its maximum position of abduction and then adduction, staying at each position for several seconds. The index finger was kept fully extended during these movements. As in Section 6.2.1, the photogrammetric method was adopted as a reference method.

**Figure 7:**
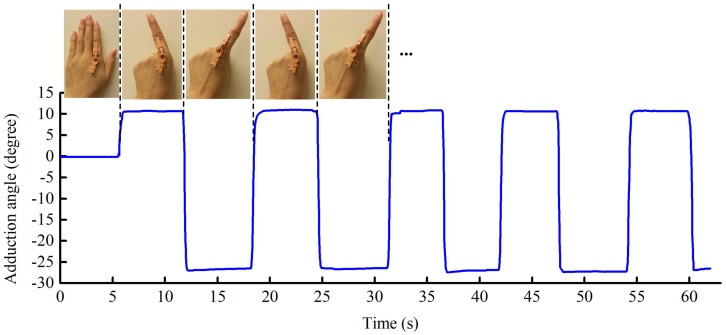
The repeated abduction and adduction of the metacarpophalangeal joint of the participant’s left index finger.

Figure 7 presents the optical sensor’s capability to track finger ABD/ADD. The readings are negative for motion left of the finger midline, and positive to the right. For the participant in this study, the measured maximum registered ABD/ADD for the left and right sides were 11.0${}^{\circ }$ and 27.5${}^{\circ }$, respectively, and this compares with readings of 12.5${}^{\circ }$ and 30.2${}^{\circ }$ obtained photogrammetrically. As seen with flexion and extension measurements, the sensing channels for ABD/ADD monitoring also have an excellent dynamic response.

#### 6.2.3. Finger Flexion/Extension and Abduction/Adduction

Simultaneous testing of finger flexion/extension and ABD/ADD was also investigated. The participant was instructed to place their hand flat on a table with fingers spread widely apart. The participant then bends the fingers into a clenched fist before extending to maximum hyperextension and ABD. This action was performed repeatedly for eight cycles.

**Figure 8:**
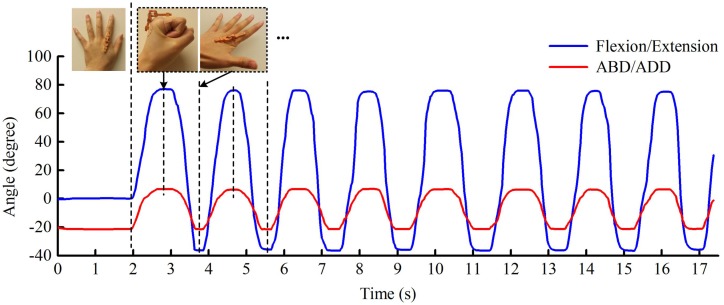
The simultaneous movements of the flexion/extension and abduction/adduction (ABD/ADD) of the metacarpophalangeal joint of the participant’s left index finger. The actions in the dotted box are repeated continuously.

The recorded finger movements are plotted in Figure 8. In this example, finger flexion co-occurring with adduction leads to increased readings. Finger extension when associated with abduction leads to falling regions in the trace. These changes are consistent with actual finger motion. The positive peak values in Figure 8 occur at the position with maximum finger flexion and adduction, i.e., clenched gesture, and the negative peaks respond to the gesture with maximum hyperextension and abduction.

During this testing, the maximum captured flexion was 77.1${}^{\circ }$. The hyperextension was up to 37.2${}^{\circ }$, much greater than the 25.1${}^{\circ }$ measured in Figure 6. This is because, in general, less hyperextension occurs when the four fingers are closed together. Additionally, the maximum ABD/ADD at the two sides are 7.1${}^{\circ }$ and 21.8${}^{\circ }$, respectively. Due to the continuous movements of the fingers, it is difficult to take photographs for comparison during this testing. However, the promising results shown in Figure 8 reveal that the optical sensor has the ability to track finger flexion/extension and ABD/ADD simultaneously.

#### 6.2.4. Finger Circumduction

The final investigation looked at finger circumduction. In this case, the participant moved their index finger to maximum hyperextension starting from the origin (0${}^{\circ }$ flexion/extension and 0${}^{\circ }$ ABD/ADD) while curling the remaining fingers inwards. The tip of the index finger was then made to execute a clockwise circular motion, which included positions of maximum ABD/ADD, i.e., circumduction.

**Figure 9:**
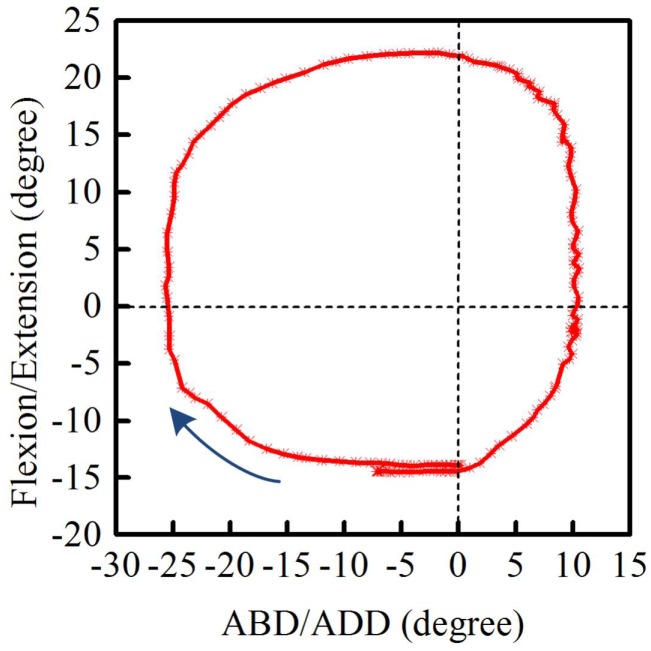
The circumduction of the metacarpophalangeal joint of the left index finger.

Figure 9 shows the clockwise trajectory of the left index finger when tracked by the developed optical sensor. During circumduction, the MCP joint exhibits four movement types. Starting from maximum hyperextension (negative reading), the MCP joint executes: flexion-ABD, flexion-ADD, extension-ABD, and extension-ADD, before returning to the initial start point. The single camera method used in this work was not capable of monitoring dynamic finger circumduction; instead, a multi-camera format is needed. Figure 9, however, suggests that this type of optical sensing has great potential for tracking finger circumduction.

In the future, we plan to investigate the performance of this two-axis sensor and compare it with time-stamped video frames for tracking dynamic finger motion.

## 7. Conclusions

An optical sensor has been developed that is capable of capturing the complex motion of the human hand. The sensor uses a two-axis measurement technique, allowing the motion of two-DOF joints to be accurately recorded. This sensor has an accuracy of ±0.3${}^{\circ }$, much higher than optical fibre and potentiometer based technologies. Additionally, a particular advantage of our sensor is its wide measurement range of 180${}^{\circ }$ which is sufficient to track the entire range of motion of human fingers. Time-consuming calibration is also unnecessary for this sensor.

When attached to the MCP joint of the index finger, the sensor has successfully tracked finger motion in real time, including flexion/extension, ABD/ADD and circumduction. The optical sensor achieved angular measurements within 2.7${}^{\circ }$ of the reference values obtained photogrammetrically. The promising results show the capability of the presented sensor for tracking two-DOF finger motion. This two-DOF optical sensor should also work for both PIP and DIP finger joints, since they have only one DOF and therefore limited mobility compared with the MCP joint. Furthermore, this sensor has the potential to be fabricated with other dimensions for monitoring other joints in the human body, e.g., ankle, wrist, and knee.

In this work, the optical sensor was attached to the hand with adhesive plasters instead of using gloves. This improves the measurement accuracy but may be unsuitable for long-term monitoring. Only the left index finger of one test subject was investigated here, so additional studies on multiple participants and multiple finger joints will be required to thoroughly validate the sensor’s reliability and reproducibility. Moreover, further reduction in size may be necessary to avoid collisions when multiple sensors are placed close to each other on the hand. These problems will be addressed in future work.

## Figures and Tables

**Table 1:** Output characteristics of the optical sensor.

Channels		Deviations from the Predicted Voltages	Hysteresis	RSD
Rotations about the *x*-axis	CH${}_{1}$	±2.4%	1.6%	0.4%
	CH${}_{3}$	±2.1%	1.4%	0.6%
Rotations about the *y*-axis	CH${}_{2}$	±2.5%	2.0%	0.6%
	CH${}_{4}$	±2.3%	1.9%	0.4%
overall		±2.3%	1.7%	0.5%

Note: CH${}_{i}$ (*i* = 1, 2, 3, and 4) represents the sensing channels of the optical sensor, and RSD denotes the relative standard deviation.
